# The protective effect of neurointerventional recanalization on the neurovascular unit in acute ischemic stroke and its correlation with serum GFAP and NfL levels

**DOI:** 10.3389/fneur.2025.1721872

**Published:** 2026-01-28

**Authors:** Ju Luo, Yang Yang, Jingmin Zhou

**Affiliations:** Department of Neurology, Huai’an Hospital Affiliated to Yangzhou University (The Fifth People’s Hospital of Huai’an), Huai’an, Jiangsu, China

**Keywords:** acute ischemic stroke, biomarkers, glial fibrillary acidic protein, mechanical thrombectomy, neurofilament light chain, neurovascular unit

## Abstract

**Aim:**

This study aimed to investigate the neuroprotective mechanisms of mechanical thrombectomy (MT) by evaluating its effects on the neurovascular unit (NVU) and correlating these effects with dynamic changes in serum biomarkers in patients with acute ischemic stroke (AIS).

**Methods:**

A prospective cohort of 128 AIS patients with anterior circulation large vessel occlusion was enrolled. Participants were divided into MT (*n* = 68) and intravenous thrombolysis (IVT) (*n* = 60) groups. Serum levels of neurofilament light chain (NfL), glial fibrillary acidic protein (GFAP), interleukin-1β (IL-1β), and tumor necrosis factor-α (TNF-α) were measured at baseline (T0), 24 h (T1), and 72 h (T2) post-treatment. Clinical outcomes included recanalization rate (mTICI grade), NIHSS improvement, and 90-day modified Rankin Scale (mRS) score.

**Results:**

The MT group showed significantly higher recanalization rates (94.1% vs. 36.7%, *p* < 0.001) and greater neurological improvement (median NIHSS improvement: 8 vs. 4, *p* < 0.001) compared to the IVT group. Serum NfL, GFAP, IL-1β, and TNF-α levels were markedly lower in the MT group at T1 and T2 (all *p* < 0.01). Strong correlations were identified between T2 NfL/GFAP levels and clinical outcomes (NIHSS improvement: *r* = −0.728/−0.663; 90-day mRS: *r* = 0.705/0.641; all *p* < 0.001).

**Conclusion:**

Successful recanalization with MT is associated with mitigated axonal injury, astrocyte activation, and neuroinflammation, findings consistent with better preservation of NVU integrity. Serum NfL and GFAP represent promising biomarkers for predicting stroke prognosis and tailoring therapeutic strategies.

## Introduction

Acute ischemic stroke (AIS) remains a major global public health challenge due to its high incidence, disability rate, and mortality. The key to treatment lies in achieving vascular recanalization within the therapeutic time window to salvage the ischemic penumbra at risk of infarction. Intravenous thrombolysis (IVT) was once the only pharmacological recanalization approach, but its efficacy for large vessel occlusion is limited. In recent years, rapid advancements in mechanical thrombectomy (MT) technology have fundamentally transformed the treatment landscape for anterior circulation large vessel occlusion-induced AIS. Numerous landmarks randomized controlled trials have confirmed that MT significantly improves vascular recanalization rates and enhances functional outcomes in patients (1, 2), establishing it as indisputable Class I clinical evidence.

However, current research predominantly focuses on the macroscopic clinical benefits of MT, such as improvements in the modified Rankin Scale (mRS) score. The underlying neuroprotective mechanisms—particularly how MT mitigates reperfusion injury and maintains cerebral microenvironmental homeostasis at the cellular and molecular levels—remain to be systematically elucidated. The previous study demonstrated that, compared with IVT alone, neurointerventional therapy combined with IVT more effectively improves cognitive function and modulates immune dysfunction in patients. This finding suggests that the therapeutic value of MT extends beyond mere mechanical revascularization and may involve a systemic biological regulation that confers benefits beyond restored blood flow (3). Transitioning from macroscopic phenotypes of “cognition and immunity” to the microstructural and functional core of the “neurovascular unit” (NVU) is an essential path for exploring its mechanistic role.

The NVU is a central concept in modern neuroscience for understanding brain function and injury. It transcends the traditional notion of the “blood–brain barrier” and represents a dynamic multicellular functional complex (4). The NVU primarily comprises vascular endothelial cells, astrocytic end-feet, pericytes, neurons, and the extracellular matrix. It regulates cerebral blood flow through sophisticated neurovascular coupling mechanisms to maintain the homeostatic microenvironment essential for neuronal survival (5).

The pathological process of AIS is essentially one of disrupted NVU integrity. Ischemia-hypoxia initially triggers energy metabolism failure, followed by activation of complex pathological mechanisms including excitotoxicity, oxidative stress, mitochondrial dysfunction, and neuroinflammatory cascades, ultimately leading to neuronal death and neural circuit disruption. It is important to note that reperfusion, while rescuing compromised tissue, may also exacerbate the aforementioned damage—a phenomenon known as “reperfusion injury” (6, 7).

In this complex pathophysiological process, the activation of astrocytes and injury to neuronal axons play pivotal roles. Astrocytes are central regulators of the NVU; upon activation, they release glial fibrillary acidic protein (GFAP), which serves both as a marker of their damage and a mediator of inflammatory regulation (8, 9). Conversely, injury to neuronal axons directly impairs neural transmission function. The release of neurofilament light chain (NfL)—a highly sensitive biomarker of axonal damage—into the cerebrospinal fluid and peripheral blood following axonal disintegration has been well-established (10, 11). Simultaneously, neuroinflammation driven by microglia and infiltrating peripheral immune cells amplifies secondary damage through the release of pro-inflammatory cytokines such as interleukin-1β (IL-1β) and tumor necrosis factor-α (TNF-α) (12, 13). These molecular events collectively determine the fate of the NVU and the ultimate neurological functional outcome of the patient.

Therefore, we hypothesize that MT, by achieving early and complete revascularization, not only rescues the ischemic penumbra but also attenuates the core drivers of NVU injury at its source, thereby exerting potent neuroprotective effects. To test this hypothesis, this study intends to employ high-sensitivity molecular detection techniques to dynamically monitor serum levels of GFAP, NfL, IL-1β, and TNF-α in AIS patients (14, 15). The aim is to accurately elucidate the protective effects of MT on the NVU and clarify the intrinsic relationship between this protection and patient neurological functional recovery. This will provide deeper biological evidence beyond recanalization rates to support the clinical application of MT and identify novel targets for future combined neuroprotective strategies.

## Materials and methods

### Study subjects and design

This study adopted a prospective cohort design and strictly adhered to the STROBE statement for reporting. Subjects were consecutively enrolled from patients with acute ischemic stroke due to large vessel occlusion in the anterior circulation who were admitted to our hospital’s stroke center between January 2023 and June 2024. The study protocol was approved by the hospital’s ethics review committee, and all patients or their legal guardians provided detailed written informed consent prior to enrollment.

Inclusion criteria were: (1) age ≥18 years; (2) clinical diagnosis of acute ischemic stroke with a clear onset-to-admission time: time window <4.5 h for those eligible for intravenous thrombolysis (IVT), and <6 h for those eligible for mechanical thrombectomy (MT) (extendable to 24 h based on multimodal imaging assessment); (3) National Institutes of Health Stroke Scale (NIHSS) score ≥6, indicating moderate to severe neurological deficit; (4) computed tomography angiography (CTA) or magnetic resonance angiography (MRA) confirmed occlusion of the intracranial segment of the internal carotid artery or the M1 segment of the middle cerebral artery; (5) absence of intracranial hemorrhage on non-contrast CT, and Alberta Stroke Program Early CT Score (ASPECTS) ≥6 or multimodal MRI-DWI-ASPECTS ≥5.

Exclusion criteria included: (1) pre-stroke severe disability (modified Rankin Scale score >2); (2) known severe cardiac, hepatic, or renal dysfunction (e.g., end-stage renal disease requiring dialysis), malignant tumors, or hematologic diseases; (3) active systemic infection on admission, definite immune system disease, or long-term use of immunosuppressants; (4) definite contraindication or allergy to contrast agents, alteplase, or study-related drugs; (5) pregnancy or lactation; (6) failure to complete the full treatment protocol or loss to follow-up.

According to the above criteria, a total of 128 patients were finally included. Based on the revascularization strategy received, all enrolled patients were divided into two groups: the MT group (receiving bridging therapy or direct thrombectomy) with 68 patients, and the IVT group (receiving standard IVT only without successful recanalization or with re-occlusion without rescue MT) with 60 patients.

*Treatment decision and group composition rationale*: It is crucial to clarify the non-randomized nature of this observational cohort. Treatment allocation (MT or IVT) was determined by a multidisciplinary stroke team based on a comprehensive assessment of admission clinical status, imaging findings (CTA/MRA, ASPECTS), eligibility per contemporary guidelines, and real-world factors such as patient/family consent and procedural availability. The MT group comprised patients who met guideline criteria for endovascular therapy and successfully underwent the procedure. The IVT group, described as receiving standard IVT without subsequent successful recanalization, represents a heterogeneous population where MT was not pursued. Primary reasons included: (a) presentation outside the institutional MT time window (e.g., early in the study period, some patients within 4.5 h but beyond 6 h for MT received only IVT); (b) the presence of relative contraindications to MT (e.g., severe vascular tortuosity, patient/guardian refusal of invasive intervention); or (c) apparent initial recanalization post-IVT (mTICI 2b-3) with subsequent re-occlusion for which rescue MT was not performed. It is important to note that no patient who achieved successful recanalization (mTICI 2b-3) with IVT alone was intentionally excluded from the MT group or retained in the IVT group for comparative purposes. The IVT group, by our study definition, exclusively comprised patients who did not achieve sustained successful recanalization (mTICI <2b) after IVT, or who experienced early re-occlusion without subsequent rescue MT. Consequently, the IVT group inherently represents a population with poor revascularization outcomes. This introduces a fundamental selection bias, as the groups compare “patients achieving successful recanalization (predominantly via MT)” with “patients having poor revascularization outcomes.” This distinction is critical for interpreting the results as an analysis of associations with revascularization status rather than a direct comparison of equivalent treatment strategies.

There were no statistically significant differences (*p* > 0.05) between the two groups in terms of age, gender, baseline NIHSS score, onset-to-puncture time (OPT)/onset-to-needle time (ONT), cardiovascular risk factors (hypertension, diabetes, atrial fibrillation, previous stroke history), or baseline ASPECTS, indicating good baseline comparability (Table 1).

**Table 1:** Comparison of baseline characteristics between two groups.

Characteristic	MT group (*n* = 68)	IVT group (*n* = 60)	*p*-value
Age (years, $\bar{x}\pm s$)	65.8 ± 10.2	67.3 ± 9.5	0.376
Male (*n*, %)	40 (58.8%)	32 (53.3%)	0.522
Baseline NIHSS [scores, M (IQR)]	16 (12–19)	15 (11–18)	0.263
Onset-to-treatment time (min, $\bar{x}\pm s$ )	245 ± 45	230 ± 50	0.082
Hypertension (*n*, %)	45 (66.2%)	38 (63.3%)	0.736
Diabetes mellitus (*n*, %)	22 (32.4%)	18 (30.0%)	0.773
Atrial fibrillation (*n*, %)	25 (36.8%)	20 (33.3%)	0.689
Previous stroke (*n*, %)	12 (17.6%)	9 (15.0%)	0.692
Baseline ASPECTS [scores, M (IQR)]	8 (7–9)	8 (7–9)	0.734

MT, mechanical thrombectomy; IVT, intravenous thrombolysis; NIHSS, National Institutes of Health Stroke Scale; ASPECTS: Alberta Stroke Program Early CT Score. Data are presented as ($\bar{x}\pm s$ ) for normally distributed continuous variables, M (IQR) for non-normally distributed variables, and *n* (%) for categorical variables.

### Treatment methods and procedures

All patients entered the stroke green channel upon admission and received standardized emergency assessment and management.

*Intravenous thrombolysis (IVT) protocol*: Eligible patients received intravenous infusion of alteplase (Boehringer Ingelheim, Germany) at a guideline-recommended dose of 0.9 mg/kg (maximum 90 mg), with 10% of the dose administered as a bolus over 1 min and the remaining 90% infused continuously over 60 min.

*Mechanical thrombectomy (MT) protocol*: Patients in the MT group were transferred to the digital subtraction angiography (DSA) room during or immediately after IVT. Under general anesthesia or conscious sedation, the femoral artery was punctured using a modified Seldinger technique, and an 8F arterial sheath was placed. After confirming the occlusion site via full cerebral angiography, an 8F guide catheter (e.g., Neuron^™^ Max, Penumbra Inc.) was superselected into the affected internal carotid or vertebral artery under roadmap guidance. A microcatheter (e.g., Velocity, Penumbra Inc.) was advanced through the thrombus over a microguidewire (e.g., Synchro-14, Stryker). Thrombectomy was then performed using stent retrieval (e.g., Solitaire^™^ X, Medtronic), direct aspiration with an aspiration catheter (e.g., ACE^™^ 68, Penumbra Inc.), or a combined SWIM technique, based on the operator’s judgment and lesion characteristics. The goal was to achieve antegrade reperfusion of mTICI grade 2b or higher. Postoperatively, blood pressure was strictly monitored and maintained below 180 mmHg systolic, with standard dual antiplatelet therapy (for stent placement) and statins for plaque stabilization.

### Observation indicators and detection methods

(1) Vascular recanalization assessment: Two senior neurointerventionists, blinded to group allocation, independently evaluated immediate post-procedural DSA images using the modified thrombolysis in cerebral infarction (mTICI) grading system: grade 0 (no perfusion), grade 1 (minimal perfusion), grade 2a (partial perfusion <50%), grade 2b (partial perfusion ≥50%), grade 3 (complete perfusion). mTICI grades 2b and 3 were defined as successful recanalization.
(2) Serum biomarker testing: Peripheral venous blood (5 mL) was collected at three fixed time points: T0 (immediately before treatment), T1 (24 ± 2 h post-procedure), T2 (72 ± 2 h post-procedure). Samples were centrifuged at 4 °C and 3,000 rpm for 15 min within 30 min of collection. The supernatant serum was separated, aliquoted into enzyme-free EP tubes, and stored uniformly at −80 °C until testing. Concentrations of four serum biomarkers were measured using enzyme-linked immunosorbent assay (ELISA), strictly following kit instructions. Human neurofilament light chain (NfL) was detected using a kit from Shanghai Enzyme-linked Biotechnology Co., Ltd. (detection range: 15.6–1,000 pg/mL, intra-assay CV <8%, inter-assay CV <10%); human glial fibrillary acidic protein (GFAP) was detected using the Quantikine ELISA kit from R&D Systems, United States (detection range: 0.156–10 ng/mL, sensitivity 0.016 ng/mL); human interleukin-1β (IL-1β) and tumor necrosis factor-α (TNF-α) were detected using kits from Shenzhen Dakewei Biotechnology Co., Ltd. (detection ranges: 4.69–300 pg/mL and 7.81–500 pg/mL, respectively). All samples were tested in duplicate, and averages were taken to reduce error.
(3) Clinical outcome assessment: Two neurologists, blinded to group allocation, assessed the National Institutes of Health Stroke Scale (NIHSS) at admission (T0) and on day 7 post-procedure (or at discharge) to evaluate neurological deficit severity. The modified Rankin Scale (mRS) was assessed at 90 ± 7 days after onset via standardized outpatient follow-up or structured telephone interview to evaluate long-term functional outcome. An mRS score of 0–2 was defined as a favorable functional outcome, and 3–6 as poor outcome.

### Statistical analysis

All statistical analyses were performed using IBM SPSS Statistics (Version 27.0). The normality of continuous data distribution was assessed using the Shapiro–Wilk test. Serum biomarker concentrations (NfL, GFAP, IL-1β, TNF-α) were found to be right-skewed; therefore, log-transformed values were used for all parametric analyses, including repeated-measures ANOVA and regression models, to meet the assumption of normality. The presented raw values [mean ± SD or median (IQR)] are for descriptive purposes only. Normally distributed continuous data are expressed as mean ± standard deviation ($\bar{x}\pm s$) and compared between groups using independent samples *t*-test; non-normally distributed continuous data are expressed as median (interquartile range) [M (IQR)] and compared using Mann–Whitney *U* test. Categorical data are expressed as number (percentage) [*n* (%)] and compared between groups using chi-square (*χ*^2^) test or Fisher’s exact test (when expected frequency <5). Repeated-measures ANOVA was used to compare dynamic trends in serum biomarkers between groups at different time points; if an interaction effect was present, simple effect analysis was performed. Pearson correlation analysis was used to analyze the correlation between serum biomarker levels and clinical scores. All statistical tests were two-tailed, and *p* < 0.05 was considered statistically significant.

In addition to the aforementioned analyses, multivariable regression models were employed to adjust for potential confounding factors and to assess independent associations. To evaluate the independent effect of treatment group on functional outcome, a binary logistic regression model was constructed with 90-day favorable outcome (mRS 0–2) as the dependent variable. The independent variables included treatment group (MT vs. IVT), age, baseline NIHSS score, baseline ASPECTS, and onset-to-treatment time. To assess the independent association between treatment group and neurological improvement, a multiple linear regression model was used with the NIHSS improvement score as the dependent variable, adjusting for the same set of covariates. To investigate the independent prognostic value of the serum biomarkers, multiple linear regression models were performed with NIHSS improvement as the dependent variable, and ordinal logistic regression models were used for the ordinal 90-day mRS score (0–6). In these models, the log-transformed serum concentrations of NfL or GFAP at T2 were included as the primary independent variable of interest, with adjustment for treatment group, age, and baseline NIHSS score. The results of these regression analyses are presented as adjusted odds ratios (aOR) with 95% confidence intervals (CI) for logistic models, and adjusted *β* coefficients with 95% CI for linear models.

## Results

### Vascular recanalization status

**Figure 1:**
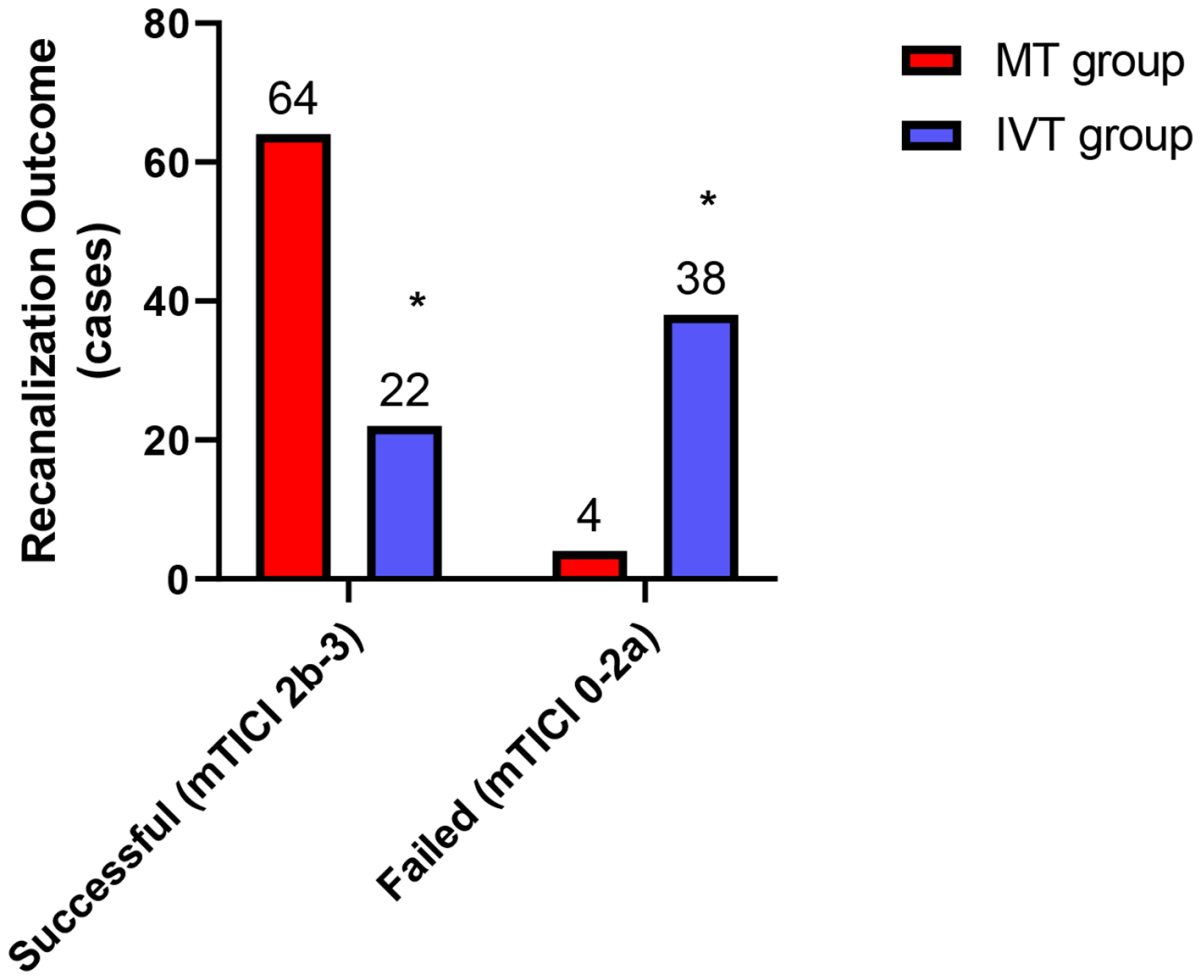
Successful recanalization rates. Bar graph comparing the rate of successful recanalization (mTICI 2b-3) between the MT group and the IVT group. The recanalization rate was significantly higher in the MT group (94.1%) than in the IVT group (36.7%) (*χ*^2^ = 48.75, ^*^p < 0.001).

Assessment of vascular recanalization revealed a significant difference in the success rate between the two groups. In the MT group, 64 out of 68 patients achieved successful recanalization (mTICI grade 2b or higher), with a recanalization rate of 94.1% (64/68). Among these, 41 cases (60.3%) reached complete recanalization (mTICI grade 3), and 23 cases (33.8%) achieved mTICI grade 2b. In contrast, in the IVT group, only 22 out of 60 patients achieved recanalization (mTICI 2b–3), resulting in a recanalization rate of 36.7% (22/60), with no cases achieving complete mTICI grade 3 recanalization (*χ*^2^ = 48.75, *p* < 0.001; Figure 1). This result reaffirms the absolute advantage of mechanical thrombectomy in recanalization for large vessel occlusion stroke, establishing a solid hemodynamic foundation for subsequent neuroprotection. Efficient and complete restoration of blood flow is a prerequisite for reducing neuronal damage in the ischemic penumbra and suppressing subsequent inflammatory cascades.

### Dynamic changes in serum biomarkers

**Figure 2:**
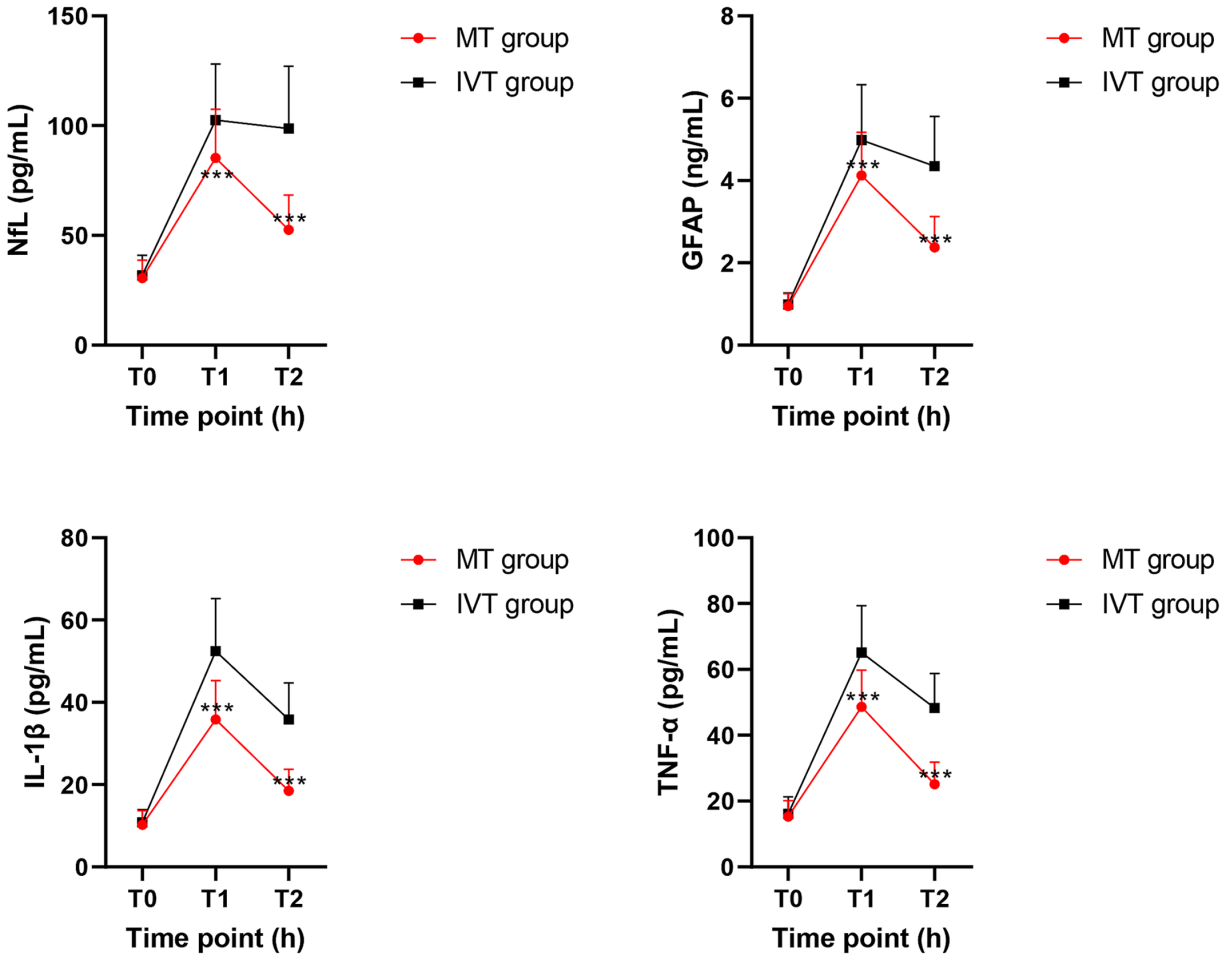
Line charts depicting the dynamic changes of serum biomarkers at T0 (pre-treatment), T1 (24 h post-procedure), and T2 (72 h post-procedure) timepoints between groups. Data are presented as mean ± SD. ^***^p < 0.01 vs. IVT group.

**Figure 3:**
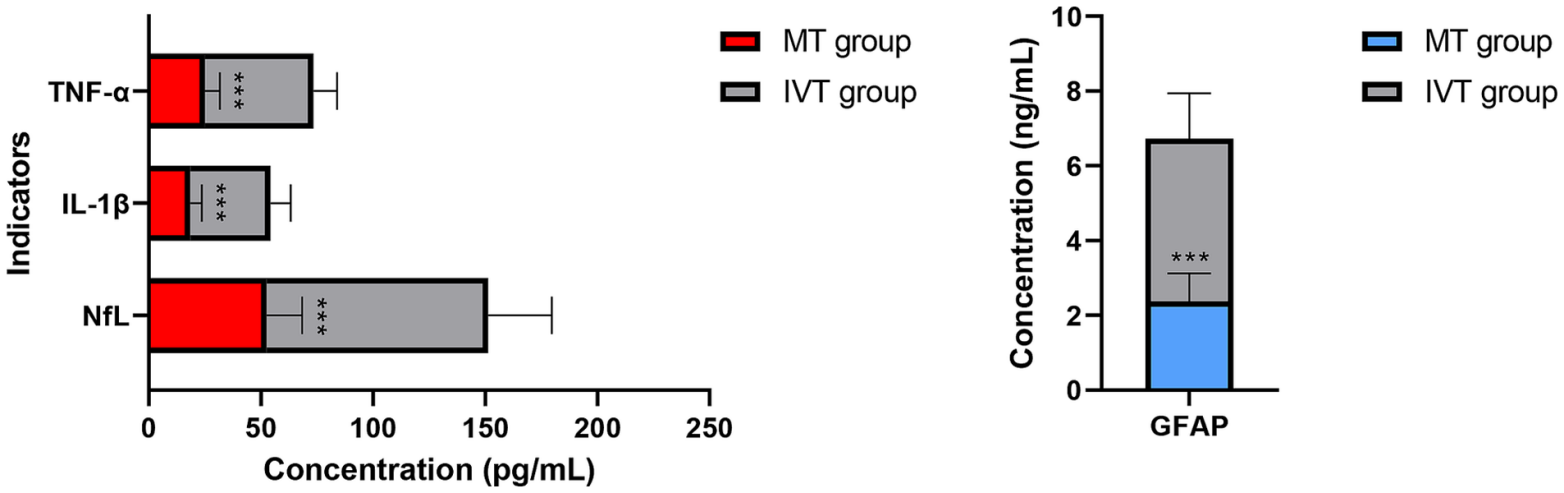
Distribution of serum biomarker levels at 72 h post-procedure (T2). Serum neurofilament light chain (NfL) levels. Serum glial fibrillary acidic protein (GFAP) levels. Concentrations are presented as box plots. Compared to the IVT group, the MT group showed significantly lower concentrations of both biomarkers (^***^p < 0.001).

Repeated-measures ANOVA indicated a significant interaction between time and group factors for the concentrations of the four serum biomarkers (NfL, GFAP, IL-1β, TNF-α) (*p* < 0.001; Table 2), suggesting distinct changing trends between the two groups across time points, as specified in Figures 2, 3.

**Table 2:** Results of repeated-measures ANOVA (serum biomarkers).

Biomarker	Time effect (*p*-value)	Group effect (*p*-value)	Time × group interaction (*p*-value)
NfL	<0.001	<0.001	<0.001
GFAP	<0.001	<0.001	<0.001
IL-1β	<0.001	<0.001	<0.001
TNF-α	<0.001	<0.001	<0.001

Repeated-measures ANOVA was used to assess differences in serum biomarker levels across timepoints (T0, T1, T2) and between groups (MT vs. IVT). A significant interaction indicates different changing trends over time between the two groups.

Within-group comparisons showed that serum NfL and GFAP concentrations in both groups peaked at 24 h post-procedure (T1), showing a significant increase compared to pre-treatment levels (T0) (all *p* < 0.01). By 72 h post-procedure (T2), concentrations of both biomarkers decreased significantly in the MT group (NfL: T1: 85.3 ± 22.1 pg/mL vs. T2: 52.6 ± 15.8 pg/mL; GFAP: T1: 4.12 ± 1.05 ng/mL vs. T2: 2.38 ± 0.74 ng/mL; *p* < 0.001), whereas the IVT group maintained near-peak levels (NfL at T2: 98.7 ± 28.4 pg/mL; GFAP at T2: 4.35 ± 1.21 ng/mL), with no significant difference from T1 (*p* > 0.05).

Between-group comparisons revealed no baseline differences at T0. At both T1 and T2 time points, serum concentrations of NfL, GFAP, IL-1β, and TNF-α in the MT group were significantly lower than those in the IVT group (*p* < 0.01). Specifically, at T2, the NfL concentration in the MT group (52.6 ± 15.8 pg/mL) was 46.7% lower than that in the IVT group (98.7 ± 28.4 pg/mL) (*t* = 11.87, *p* < 0.001); the GFAP concentration in the MT group (2.38 ± 0.74 ng/mL) was 45.3% lower than that in the IVT group (4.35 ± 1.21 ng/mL) (*t* = 10.45, *p* < 0.001). The pro-inflammatory cytokines IL-1β and TNF-α showed highly consistent trends, with significantly lower concentrations in the MT group at T2 compared to the IVT group (IL-1β: 18.5 ± 5.2 pg/mL vs. 35.8 ± 8.9 pg/mL; TNF-α: 25.1 ± 6.7 pg/mL vs. 48.3 ± 10.5 pg/mL; both *p* < 0.001). This series of data clearly demonstrates that successful mechanical recanalization effectively mitigates post-ischemic neuroglial injury and activation and significantly downregulates systemic inflammatory response levels.

### Clinical outcomes

**Figure 4:**
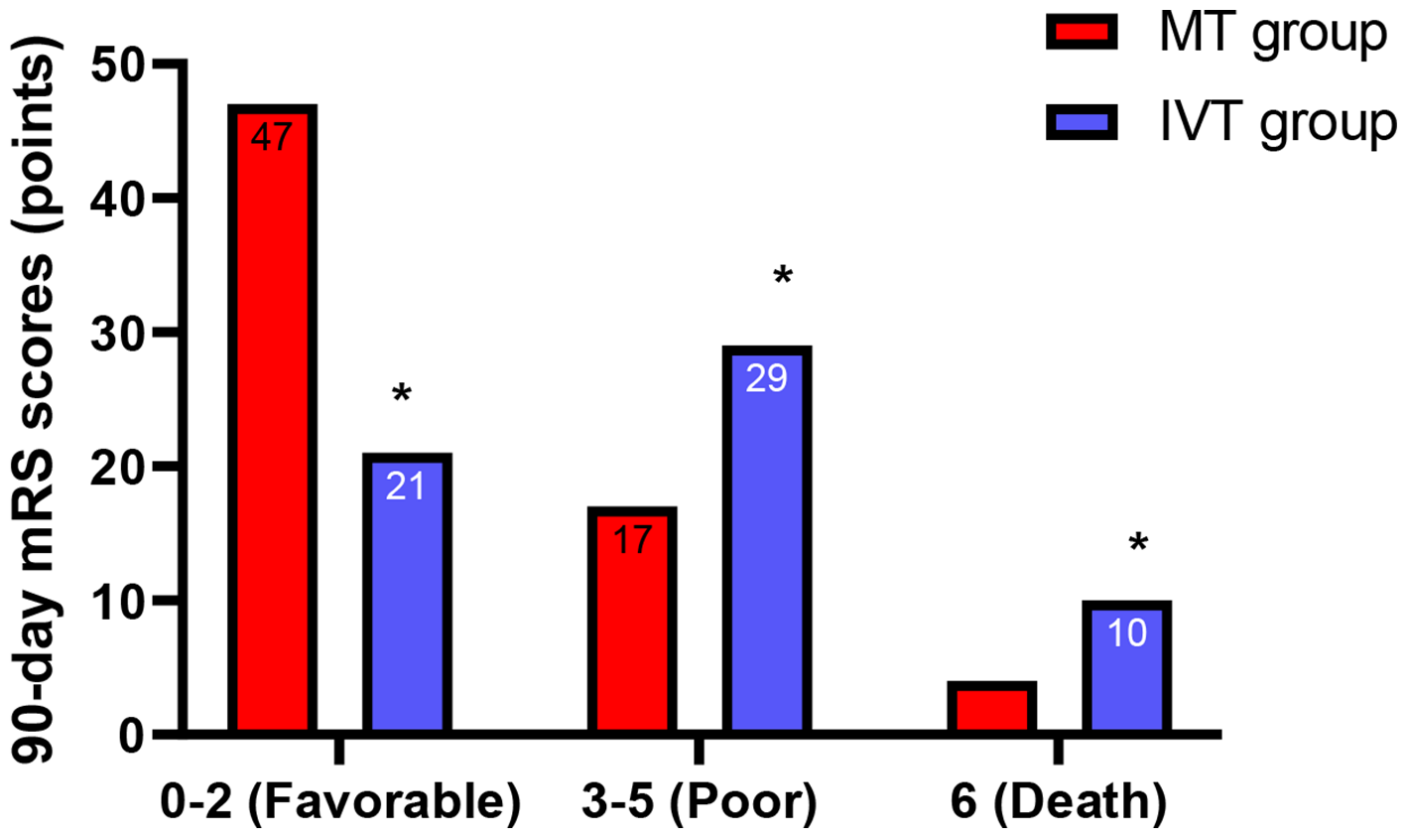
Functional outcomes at 90 days. Bar graph showing the proportion of patients achieving a favorable functional outcome (modified Rankin Scale score 0–2) at 90 days. The proportion was significantly higher in the MT group (69.1%) than in the IVT group (35.0%) (*χ*^2^ = 14.32, ^*^p < 0.001).

Neurological assessment at 7 days post-procedure showed that the median improvement in NIHSS score (admission score minus 7-day score) in the MT group was 8 (IQR: 5–11), significantly better than the 4 (IQR: 2–6) in the IVT group (*Z* = −5.892, *p* < 0.001; Table 3). Follow-up results of 90-day functional outcomes indicated that 47 patients in the MT group achieved a favorable functional outcome (mRS 0–2), accounting for 69.1% (47/68), while only 21 patients (35.0%, 21/60) in the IVT group reached a favorable outcome. The proportion of favorable outcomes in the MT group was 1.97 times that of the IVT group (*χ*^2^ = 14.32, *p* < 0.001). Additionally, the mortality rate (mRS = 6) in the MT group was 5.9% (4/68), lower than the 16.7% (10/60) in the IVT group (*χ*^2^ = 4.12, *p* = 0.042), as specified in Figure 4. These results confirm from a clinical endpoint perspective that MT treatment provides patients with significant and clinically meaningful neurological recovery, reducing the burden of disability.

**Table 3:** NIHSS improvement [M (IQR)].

Group	NIHSS score improvement
MT group	8 (5–11)
IVT group	4 (2–6)

The degree of neurological improvement was significantly greater in the MT group (*Z* = −5.892, *p* < 0.001).

### Imaging outcomes (follow-up ASPECTS)

To approximate the extent of final infarction, follow-up non-contrast CT scans performed at 24–48 h post-treatment were available for 115 patients (MT: *n* = 62; IVT: *n* = 53). The follow-up ASPECTS was assessed by two blinded neuroradiologists. The MT group showed a significantly higher median follow-up ASPECTS compared to the IVT group [8 (IQR: 7–9) vs. 5 (IQR: 4–7), *p* < 0.001]. This suggests that successful recanalization via MT was associated with less extensive parenchymal injury on early follow-up imaging, providing contextual support for the observed differences in serum biomarker levels.

### Correlation analysis

**Figure 5:**
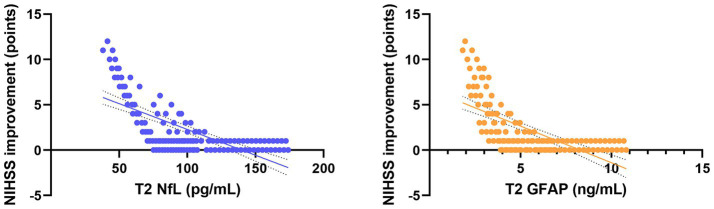
Scatter plots showing the correlation between serum NfL and GFAP levels at T2 and neurological improvement in all patients. Trend lines indicate significant negative and positive correlations.

**Figure 6:**
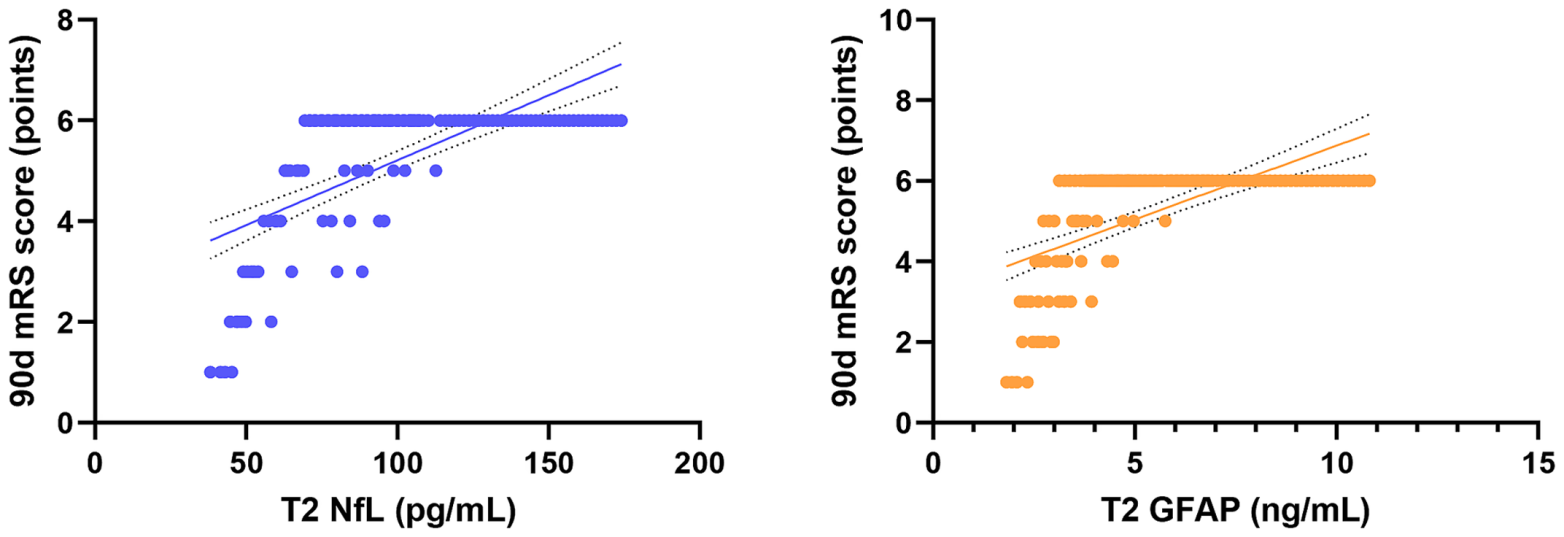
Scatter plots showing the correlation between serum NfL and GFAP levels at T2 and long-term disability in all patients. Trend lines indicate significant negative and positive correlations.

Pearson correlation analysis revealed strong associations between serum biomarkers and clinical outcomes (Table 4). In the combined analysis of all 128 patients, serum NfL and GFAP levels at 72 h post-procedure (T2) were significantly negatively correlated with the improvement in NIHSS score at 7 days post-procedure (NfL: *r* = −0.728, *p* < 0.001; GFAP: *r* = −0.663, *p* < 0.001; Figure 5). More importantly, NfL and GFAP levels at T2 were significantly positively correlated with the 90-day mRS score (NfL: *r* = 0.705, *p* < 0.001; GFAP: *r* = 0.641, *p* < 0.001; Figure 6), indicating that higher biomarker levels were associated with greater long-term disability. This finding has potential clinical guidance value: early detection (within 72 h) of serum NfL or GFAP concentrations may serve as an objective and quantitative biological indicator for assessing stroke severity and predicting long-term functional outcomes, providing valuable reference for clinicians to adjust treatment strategies and rehabilitation plans.

**Table 4:** Correlation analysis between serum biomarkers and clinical outcomes (Pearson).

Serum biomarker	Correlation with NIHSS improvement (*r*)	*p*-value	Correlation with 90 days mRS score (*r*)	*p*-value
NfL (pg/mL)	−0.728	<0.001	0.705	<0.001
GFAP (ng/mL)	−0.663	<0.001	0.641	<0.001

*n* = 128. All correlations were highly statistically significant (*p* < 0.001). A negative *r*-value indicates worse neurological improvement with higher biomarker levels; a positive *r*-value indicates greater disability.

### Multivariable and incremental prognostic value analyses

To adjust for potential confounders and to evaluate the independent and incremental prognostic value of serum biomarkers, a series of multivariable regression models were constructed.

Binary logistic regression models were built in a hierarchical manner (Table 5). Model 1 (clinical baseline model): Included established clinical predictors: age, baseline NIHSS score, and baseline ASPECTS. This model showed good discriminative ability (C-statistic = 0.79, 95% CI: 0.71–0.86). Model 2 (adding recanalization status): Successful recanalization (mTICI 2b-3) was added to Model 1. This significantly improved model fit (likelihood ratio test *χ*^2^ = 21.4, *p* < 0.001) and discrimination (C-statistic = 0.86, 95% CI: 0.79–0.92). Model 3a & 3b (adding biomarkers): Log-transformed serum NfL (Model 3a) or GFAP (Model 3b) concentration at T2 was added to Model 2. The inclusion of either biomarker led to a further significant improvement in model fit (for NfL: *χ*^2^ = 9.8, *p* = 0.002; for GFAP: *χ*^2^ = 6.1, *p* = 0.014) and increased the C-statistic (Model 3a: 0.89, 95% CI: 0.83–0.95; Model 3b: 0.88, 95% CI: 0.81–0.94). In these final models, higher log(NfL) and log(GFAP) levels remained independently associated with a lower likelihood of a favorable outcome [NfL: adjusted odds ratio (aOR) = 0.41 per log-unit increase, 95% CI: 0.24–0.69, *p* = 0.001; GFAP: aOR = 0.55, 95% CI: 0.34–0.88, *p* = 0.013], after controlling for all other covariates.

**Table 5:** Hierarchical multivariable logistic regression models for predicting 90-day favorable outcome (mRS 0–2).

Model & variables	Adjusted odds ratio (aOR)	95% confidence interval	*p*-value	Model C-statistic (95% CI)
Model 1: Clinical baseline				0.79 (0.71–0.86)
Age (per year increase)	0.98	0.94–1.02	0.305	
Baseline NIHSS (per point increase)	0.85	0.78–0.92	<0.001	
Baseline ASPECTS (per point increase)	1.32	1.01–1.72	0.042	
Model 2: Model 1 + recanalization				0.86 (0.79–0.92)
Successful recanalization (mTICI 2b-3)	4.25	1.95–9.25	<0.001	
(Variables from Model 1 omitted for clarity)				
Model 3a: Model 2 + Biomarker (NfL)				0.89 (0.83–0.95)
log(serum NfL at T2) (per log-unit)	0.41	0.24–0.69	0.001	
(Variables from Model 2 omitted for clarity)				
Model 3b: Model 2 + Biomarker (GFAP)				0.88 (0.81–0.94)
log(serum GFAP at T2) (per log-unit)	0.55	0.34–0.88	0.013	

Similar results were observed using multiple linear regression. After adjusting for age, baseline NIHSS, ASPECTS, and recanalization status, higher log(NfL) and log(GFAP) at T2 were independent predictors of less neurological improvement [log(NfL): adjusted *β* = −2.85, 95% CI: −4.10 to −1.60, *p* < 0.001; log(GFAP): adjusted *β* = −2.15, 95% CI: −3.35 to −0.95, *p* = 0.001].

These analyses demonstrate that the prognostic information carried by serum NfL and GFAP at 72 h is not redundant with, but adds significant value to, standard clinical and imaging predictors as well as the crucial factor of recanalization success itself.

## Discussion

This study dynamically monitored serum biomarkers to explore the neuroprotective mechanisms of MT from the perspective of NVU protection. Three main findings were identified: First, peak levels of serum neuronal injury markers (NfL, GFAP) and inflammatory factors (IL-1β, TNF-α) after the procedure were significantly lower in the MT group and declined more rapidly. Second, the 90-day neurological outcomes were significantly better in the MT group than in the intravenous thrombolysis (IVT)-only group. Finally, early serum NfL and GFAP levels showed strong correlations with the degree of neurological improvement and long-term prognosis. These results collectively indicate that successful recanalization achieved by MT is associated with a biomarker profile consistent with better preservation of the NVU, characterized by lower levels of secondary neural injury and inflammatory markers, which in turn correlate with improved clinical outcomes. Importantly, these associations—between treatment group and superior outcomes, and between elevated biomarkers and worse prognosis—remained statistically significant in multivariable regression models adjusting for key baseline covariates (age, baseline NIHSS, etc.), reinforcing the robustness of our primary findings.

The robust association between successful MT and attenuated biomarker levels, which persisted after multivariable adjustment, leads us to consider the underlying mechanisms. The integrity of the NVU is central to maintaining cerebral homeostasis. Ischemia-hypoxia and subsequent reperfusion injury can trigger a series of complex pathological cascades, in which astrocyte activation and neuronal axon damage are key components. This study is the first to simultaneously observe dynamic changes in specific molecular markers reflecting these processes within the same cohort. Serum NfL, a direct product of neuronal axonal cytoskeleton disintegration, sensitively reflects the severity and extent of axonal damage (16).

The significantly lower NfL levels at 72 h post-procedure in the MT group are consistent with the establishment of successful reperfusion and are likely reflective, in large part, of a reduction in final infarct volume. A smaller infarct core inherently releases less axonal cytoskeleton protein. This interpretation is supported by our imaging findings, which showed that the MT group had significantly higher follow-up ASPECTS scores (median 8 vs. 5, *p* < 0.001), indicating better preservation of brain parenchyma and thus, a smaller effective infarct burden. Beyond this primary mechanism of limiting infarct growth, the observed kinetic profile—lower and more rapidly declining NfL—could be consistent with a moderation of ongoing Wallerian degeneration in the salvaged penumbra, thereby better preserving the structural integrity of white matter tracts. This interpretation aligns with a recent basic study showing that reperfusion rapidly alleviates calcium overload in axonal mitochondria, thereby inhibiting calpain-mediated neurofilament proteolysis (17).

Meanwhile, the marked reduction in GFAP, a marker of astrocyte activation, is equally significant and follows a similar explanatory framework: successful recanalization leading to reduced infarct size results in less extensive reactive astrogliosis. Astrocytes act as “commanders” of the NVU, with their end-feet enveloping blood vessels and regulating blood–brain barrier permeability and cerebral blood flow (18, 19). The attenuated rise and more rapid decline of GFAP in the MT group are compatible with the notion that efficient recanalization is associated with a reduction in the drivers of cytotoxic edema and reactive astrogliosis, helping maintain normal supportive and regulatory functions and thus stabilizing the NVU microenvironment.

Notably, the concentrations of pro-inflammatory factors IL-1β and TNF-α were also significantly lower in the MT group, suggesting an association between successful MT and a modulated systemic inflammatory response. After cerebral ischemia, damaged neurons and glial cells release large amounts of damage-associated molecular patterns (DAMPs), activating the innate immune system and leading to microglial activation, infiltration of peripheral neutrophils and monocytes/macrophages, and a “cytokine storm,” which further disrupts the blood–brain barrier and induces neuronal apoptosis (20, 21). The observed attenuation of inflammation post-MT may stem from two aspects: first, the removal of ischemic core tissue that continuously releases DAMPs, eliminating the source of sustained inflammatory activation; second, rapid restoration of blood flow clears locally accumulated pro-inflammatory mediators, interrupting the positive feedback loop of inflammation. This dovetails perfectly with previous finding that “MT improves immune function” in patients (22), extending from macroscopic immune regulation to microscopic molecular mechanisms.

The most clinically significant finding of this study is the highly significant correlation between serum NfL and GFAP levels and early neurological improvement (NIHSS change) as well as long-term disability (90-day mRS). This indicates that these biomarkers are not merely bystanders in the pathological process but are key indicators reflecting the progression of neural damage and repair. For instance, persistently high NfL levels suggest ongoing axonal injury and may predict poor outcomes (23). This highlights a potential future direction in stroke treatment: dynamic monitoring of serum NfL/GFAP could potentially serve as a “liquid biopsy” tool for assessing treatment response and predicting prognosis, enabling earlier individualized patient management. For patients with successful recanalization but persistently elevated biomarker levels, microcirculatory disturbance or secondary injury may be present, warranting more aggressive neuroprotective or anti-inflammatory interventions.

The strength and independence of these associations were further substantiated through multivariable and incremental value analyses. As detailed in the results (Table 5), hierarchical regression modeling was employed. First, a baseline clinical model incorporating age, baseline NIHSS, and baseline ASPECTS was established. The addition of successful recanalization status significantly improved this model. Crucially, when log-transformed serum NfL or GFAP levels at 72 h were added, they provided significant independent prognostic value, further enhancing the model’s ability to discriminate between patients with good and poor 90-day outcomes (increase in C-statistic). This demonstrates that the prognostic information carried by these early biomarkers is not redundant with, but rather complementary to, both the initial clinical presentation and the critical treatment milestone of successful reperfusion.

Certainly, this study has several limitations. First, and most importantly, this study has inherent limitations due to its observational design and significant selection bias. As elaborated in the Methods, the IVT group was not a randomly assigned control but was formed based on complex real-world clinical decisions and, by definition, comprised patients with unsuccessful or incomplete revascularization (mTICI <2b or re-occlusion). Therefore, the observed advantages of the MT group in biomarker profiles and clinical outcomes are intrinsically conflated with the success of revascularization itself. The IVT group represents a non-recanalized/partially recanalized population, which fundamentally limits our ability to draw causal conclusions about specific neuroprotective properties of MT independent of its revascularization efficacy. Thus, our findings should be interpreted as demonstrating that ‘successful early revascularization (achieved primarily by MT in this cohort) is associated with attenuated levels of injury biomarkers, reduced inflammation, and better functional outcomes, likely mediated through a reduction in final infarct volume,” rather than providing definitive causal evidence for neuroprotective effects of MT independent of reperfusion. The biomarker patterns are consistent with better preservation of the NVU in the context of successful reperfusion. Future randomized controlled trials are needed to confirm a causal mechanistic role for MT in NVU protection. Second, a major methodological limitation is the lack of systematic data on final infarct volume. As astutely noted by the reviewer, the most parsimonious explanation for the lower biomarker levels in the MT group is the establishment of successful reperfusion leading to a smaller infarct. Without quantifying infarct volume on follow-up imaging (e.g., 24–72 h MRI-DWI or FLAIR), we cannot statistically disentangle the treatment effect on biomarker levels from its effect on infarct size. We were therefore unable to perform mediation analyses to determine to what extent the biomarker benefits were direct or mediated through infarct volume reduction, nor could we adjust for infarct volume in regression models to identify potential independent effects. This omission precludes a more refined mechanistic interpretation and underscores that our findings primarily associate successful recanalization with a biomarker profile characteristic of less extensive brain infarction. Future studies integrating serial biomarker measurement with precise volumetric infarct analysis are essential to delineate these relationships more clearly. Third, only peripheral blood biomarkers were measured; although they have been shown to correlate well with central nervous system injury, simultaneous cerebrospinal fluid analysis could provide more direct central evidence. Finally, the neurological assessment did not include more detailed cognitive domain evaluations, preventing a more direct linkage between micro-level changes and the macro-level cognitive outcomes observed in our previous research.

## Conclusion

This observational study demonstrates that successful early recanalization, predominantly achieved by mechanical thrombectomy in this cohort, is strongly associated with a favorable multi-dimensional profile: superior vascular reperfusion, attenuated serum levels of injury and inflammatory biomarkers (NfL, GFAP, IL-1β, TNF-α), and significantly improved neurological and functional outcomes at 90 days. The marked reduction in axonal and glial injury biomarkers likely reflects, as a principal mechanism, the crucial consequence of successful reperfusion—a reduction in final infarct volume. The strong correlations observed between early post-procedural serum NfL/GFAP levels and both short-term neurological improvement and long-term disability highlight the potential of these biomarkers as objective, dynamic tools for prognostic stratification and possibly for monitoring therapeutic response. While the biomarker patterns are consistent with the concept of neurovascular unit preservation in the context of effective reperfusion, future studies incorporating randomized designs and systematic infarct volumetry are needed to delineate causal pathways and distinguish the effects of reperfusion per se from other potential neuroprotective mechanisms. Nonetheless, these findings reinforce the central importance of achieving rapid and complete recanalization and support the further investigation of serum NfL and GFAP as complementary biomarkers in stroke management.

## Data Availability

The original contributions presented in the study are included in the article/supplementary material, further inquiries can be directed to the corresponding author.
